# Extended Trochanteric Osteotomy with Intermediate Resection Arthroplasty Is Safe for Use in Two-Stage Revision Total Hip Arthroplasty for Infection

**DOI:** 10.3390/jcm11010036

**Published:** 2021-12-22

**Authors:** Sebastian Hardt, Vincent Justus Leopold, Thilo Khakzad, Matthias Pumberger, Carsten Perka, Christian Hipfl

**Affiliations:** Centre for Musculoskeletal Surgery, Department of Orthopaedics, Charité Universitaetsmedizin Berlin, Charitéplatz 1, 10117 Berlin, Germany; sebastian.hardt@charite.de (S.H.); vincent.leopold@charite.de (V.J.L.); thilo.khakzad@charite.de (T.K.); matthias.pumberger@charite.de (M.P.); carsten.perka@charite.de (C.P.)

**Keywords:** extended trochanteric osteotomy, revision total hip arthroplasty, periprosthetic infection, two-stage revision, resection arthroplasty, reinfection

## Abstract

Background: This study sought to compare the results of two-stage revision total hip arthroplasty (THA) for periprosthetic infection (PJI) in patients with and without the use of an extended trochanteric osteotomy (ETO) for removal of a well-fixed femoral stem or cement. Methods: Thirty-two patients who had undergone an ETO as part of a two-stage revision without spacer placement were matched 1:2 with a cohort of sixty-four patients of the same sex and age who had stem removal without any osteotomy. Clinical outcomes including interim revision, reinfection and aseptic failure rates were evaluated. Modified Harris hip scores (mHHS) were calculated. Minimum follow-up was two years. Results: Patients undergoing ETO had a significantly lower rate of interim re-debridement compared to non-ETO patients (0% vs. 14.1%, *p* = 0.026). Reinfection following reimplantation was similar in both groups (12.5% in ETO patients vs. 9.4% in non-ETO patients, *p* = 0.365). Revision for aseptic reason was necessary in 12.5% in the ETO group and 14.1% in the non-ETO group (*p* = 0.833). Periprosthetic femoral fractures were seen in three patients (3.1%), of which all occurred in non-ETO patients. Dislocation was the most common complication, which was equally distributed in both groups (12.5%). The mean mHHS was 37.7 in the ETO group and 37.3 in the non-ETO group, and these scores improved significantly in both groups following reimplantation (*p* < 0.01). Conclusion: ETO without the use of spacer is a safe and effective method to manage patients with well-fixed femoral stems and for thorough cement removal in two-stage revision THA for PJI. While it might reduce the rate of repeated debridement in the interim period, the use of ETO appears to lead to similar reinfection rates following reimplantation.

## 1. Introduction

Periprosthetic joint infection (PJI) remains one of the most challenging complications to manage in the field of arthroplasty [1,2,3,4]. Given the increasing prevalence of PJI, which is associated with significant morbidity and health-care costs, and the clinically poor outcomes after a failed revision, a standardized therapeutic approach is critical for its management [5].

The most widely used surgical strategy for hip PJI is a two-stage exchange arthroplasty with a temporary antibiotic-loaded cement spacer [6,7,8,9,10,11,12]. In the setting of a well-fixed femoral component or bone cement mantle, an extended trochanteric osteotomy (ETO) is a useful adjunct to simplify the procedure during the first-stage surgery so as not to further compromise the existing bone stock which may complicate further reconstruction [13,14,15,16,17]. Further advantages of ETO in cases of PJI are the excellent exposure of the proximal femur and easier feasibility of radical debridement of the bone–cement interface [18,19,20,21,22]. The use of ETO in two-stage revision for infected total hip arthroplasty (THA) has shown excellent union rates and comparable infection-free survival rates [18,19,20,21,22]. However, in the setting of ETO and/or bone loss, spacer-associated complications such as spacer dislocation, spacer migration, spacer breakage and femur fracture are not uncommon [23,24,25,26,27,28,29]. Therefore, we performed ETOs in cases with bone defects and/or abductor deficiency without interim spacers to avoid such complications. The major concerns of this surgical strategy are compromising infection eradication without having the local antibiotic effects of the spacer and hampering reimplantation due to resulting muscle contractures ultimately leading to worse functional outcomes [30]. However, the local antimicrobial effects of the spacer do not appear to play a significant role in infection eradication [31], and comparable functional results are achievable when long interim periods are avoided [32,33,34]. To date, only a few clinical studies have been reported regarding the use of ETO in the setting of two-stage revision THA with varying surgical regimens [18,19,20,21,22] and no study has specifically investigated the safety of ETO without spacer placement.

For this reason, the purpose of this study was to analyze the clinical, functional and radiological outcomes of patients who received an ETO in a nonspacer two-stage exchange THA.

## 2. Materials and Methods

### 2.1. Study Design

Using our prospectively maintained institutional database, we identified all consecutive patients who underwent an entire two-stage exchange arthroplasty for periprosthetic hip infection from January 2013 to August 2017 with the use of an ETO to remove a well-fixed femoral component or femoral cement mantle (ETO group: 32 hips/32 patients). The control group consisted of patients who underwent a two-stage exchange arthroplasty without the use of an ETO, any femoral osteotomy or femoral cortical window during the same period and was matched for age and sex at a 1:2 ratio (non-ETO group: 64 hips/64 patients) to aim for a more homogenous setting for comparison of outcome parameters. No spacer was used in either group to prevent spacer-related complications. The individual decision to perform an ETO was based on the preoperative radiographs or intraoperative situation. Patients with a well-fixed cementless stem after multiple frustrated attempts of endofemoral removal with conventional techniques as well as patients with cemented stems with a well-interdigitated cement mantle were indicated for ETO. Patients with any violation of the standardized treatment protocol and follow-up less than 24 months were excluded. Surgical and antimicrobial treatment was performed according to a standardized algorithm by a multidisciplinary team of orthopedic surgeons, infectious disease physicians and microbiologists [1,3,35].

### 2.2. Diagnosis of PJI

PJI was diagnosed based on the definition reported by Zimmerli et al. [1,36], which included the confirmation of at least one of the following criteria: sinus tract or purulence around the prosthesis; increased synovial white blood cell (WBC) count or polymorphnuclear (PMN) percentage (>2000/µL WBC or >70% PMN); confirmatory microbial growth in synovial fluid, periprosthetic tissue (≥1 specimen in highly virulent pathogens or ≥2 specimens in low virulent pathogens) or sonication culture of retrieved prosthesis components (>50 colony-forming units (CFU)/mL sonication fluid [37]); or positive histopathology, defined as a mean of ≥23 granulocytes per 10 high-powered fields [38].

### 2.3. Surgical Technique

**Figure 1:**
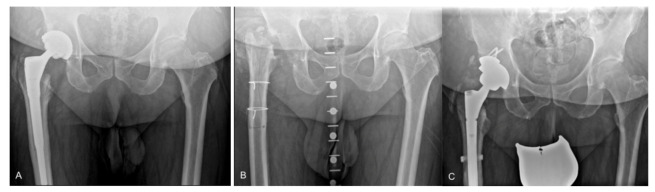
(**A**) Anteroposterior radiograph of a 68-year-old male patient with culture negative periprosthetic infection of the right hip showing a well-fixed cementless long stem 6 years after implantation. (**B**) Radiograph taken after first-stage total hip arthroplasty (THA) removal using an extended trochanteric osteotomy (ETO) with cerclage fixation without spacer insertion. (**C**) Postoperative radiograph showing the reimplantation after 7 weeks utilizing a highly porous metal shell with augment and modular fluted tapered stem. At the time of reimplantation, ETO was successfully healed and cerclages were exchanged.

All operations were performed by five senior surgeons specialized in total joint arthroplasty with experience in revision THA. During the first-stage surgery, a complete removal of all prosthetic components, cement, plug and all other foreign material was performed. The ETO was performed based on the principles originally described by Wagner [13] and later popularized by Younger et al. [14]. In 24 patients (75%), in whom the prior exposure was an anterolateral approach, ETO was done via an anterolateral approach, and in 8 cases (25%) a distally extended posterolateral approach was used. The femoral osteotomy was performed directly anterolateral to the linea aspera and includes the entire greater trochanter and part of the femoral diaphysis. The length of the bony window depended on the length of the inserted prosthesis or femoral cement mantle and was planned based on the preoperative radiographs (Figure 1A). In order to maintain vascularity to the osteotomy fragment, we minimized stripping of the vastus lateralis from the fragment, while preserving the attachments of the abductor. After removal of the femoral component and/or cement, radical debridement of the proximal femur was performed and the ETO fragment was then fixed with 2–4 cerclages depending on the osteotomy length and fragment stability. Synovial fluid was aspirated, if available, and five periprosthetic tissue samples were obtained for microbiological analysis. The retrieved prosthetic components were sent for sonication. A thorough irrigation and debridement was then performed using a polyhexanide-containing solution. The wound was closed routinely in layers over a passive drain without the use of a spacer (Figure 1B).

Second-stage reimplantation was carried out, when the local status was satisfactory (no drainage, redness or increased swelling), laboratory signs of infection control (decreasing C-reactive protein) were present and the general status of the patient was appropriate. Any signs of infection persistence prompted an interim re-debridement including exchange of the cerclages. The decision to perform a re-debridement was based on clinical features, laboratory parameters and intraoperative findings. Surgeries performed for wound coverage were not considered as interim re-debridement.

During reimplantation, the ETO was assessed intraoperatively and, if it was healed or firm both clinically and radiologically, the cerclages were exchanged (*n* = 30) or removed without replacement (*n* = 2). If the osteotomy was unstable, the cerclages were first removed, then the window was reopened again for stem implantation (*n* = 9). A prophylactic cerclage was placed around the isthmus prior to stem implantation in 9 cases (28%). After stem implantation, the ETO was closed using cerclages. Reimplantation was predominantly performed using cementless implants (Figure 1C). All operative characteristics are summarized in Table 1.

### 2.4. Antimicrobial Treatment

After the first-stage surgery, broad-spectrum, intravenous antibiotics were administered for two weeks followed by oral antibiotics until reimplantation. Oral antibiotics were chosen according to susceptibility testing, oral bioavailability, and osseous penetration. Details of all microorganisms are shown in Table 2. Between stages, all patients received ongoing antimicrobial treatment. No drug holidays or diagnostic hip aspiration was performed prior to second-stage surgery [39].

After second-stage reimplantation, patients were treated with intravenous antibiotics for two weeks followed by oral antibiotics for four weeks. In the case of significant microbiological results at reimplantation (≥2 positive cultures, polymicrobial growth, or ≥1 positive culture, if the pathogen was the same as the initial infecting microorganism or a new highly virulent pathogen), antimicrobial treatment was extended from six to twelve weeks postoperatively.

### 2.5. Radiographic Analysis

Radiographic analysis was conducted by an orthopedic surgeon specializing in hip arthroplasty and an orthopedic surgery resident for all anteroposterior and lateral hip radiographs. Femoral and acetabular bone loss was classified according to Paprosky et al. [40] and Valle and Paprosky [41], respectively. Preoperative and postoperative radiographs were analyzed to assess ETO length, ETO migration, ETO healing, femoral component subsidence, leg-length and offset. ETO union was determined by the presence of callus bridging and/or disappearance of the osteotomy line. ETO fragment migration was measured as the change in the distance from the first cable above the osteotomy site and the tip of the greater trochanter. Femoral stem subsidence was measured as the change in the distance from the most proximal point on the lesser trochanter to the center of the femoral head. Leg-length difference was measured via the difference between the interteardrop line and the lesser tubercle line. Offset difference was determined as the difference between the THA offset and contralateral offset.

### 2.6. Outcome Measures

Primary outcome measures included revisions performed for reinfection and aseptic reasons, revisions for any reason and complications. Reinfections were identified together with infectious disease physicians. Functional outcome was analyzed for all patients alive who did not had revision surgery following reimplantation, calculating the modified Harris Hip Score (mHHS) [42].

### 2.7. Statistical Analysis

Descriptive statistics are presented as numbers (percentage) and means (range). Mann-Whitney U test and chi-square test were used to compare continuous variables and categorical variables, respectively. Kaplan-Meier survival estimates were calculated by using revision for reinfection and revision for any reason as an end point. The log-rank test was used for survival comparison between the ETO and non-ETO group. Calculations were performed using SPSS version 25 software (SPSS Inc., Chicago, IL, USA). A *p*-value less than 0.05 was considered statistically significant.

## 3. Results

### 3.1. Demographics

The ETO group consisted of 21 females and 11 males with a mean age of 71 years (range: 46–88 years) and the mean body mass index (BMI) was 27 kg/m^2^ (range: 20–41 kg/m^2^). A total of 12 patients (38%) had prior revision for infection and 8 patients (25%) had a sinus tract. Besides the matched parameters, there were no significant differences in baseline demographics between the ETO and non-ETO group (Table 3). The average prosthesis-free interval was 8.8 weeks (range: 2.7–29 weeks) and 9.1 weeks (range: 2.0–24.9) in the ETO group and non-ETO group, respectively (*p* = 0.712). Patients undergoing ETO had an increasingly severe femoral bone loss (*p* = 0.001), a longer duration of both surgeries (*p* = 0.0001 and *p* = 0.046, respectively), and reimplantation was more frequently performed with modular fluted tapered stems (*p* = 0.0001). Other operative characteristics were similar in the two groups (Table 1). Two patients died in the ETO group and four patients in the non-ETO group at the point of the latest follow-up. The mean follow-up was 5.5 years (range: 3.2–7.5 years) and 5.5 years (range: 3.0–7.7 years) in the ETO and non-ETO group, respectively.

### 3.2. Microbiology and Reinfection

No significant differences were found in the microorganism frequency in both the first-stage and second-stage surgery. In both groups, the most common microorganism was coagulase-negative Staphylococcus followed by Staphylococcus aureus and Cutibacterium acnes. All pathogens leading to PJI are summarized in Table 2. Positive cultures at the time of reimplantation were found in three (9%) of the patients with ETO and six (9%) of the patients without ETO.

A total of nine patients underwent re-debridement in the interim period. Patients with ETO had a significantly lower risk of repeated debridement between the two stages compared to patients without ETO (0% vs. 14%; *p* = 0.026). We found no significant difference in the reinfection rates after second-stage reimplantation between the two groups. Reinfection occurred in four (13%) of the patients with ETO and six (9%) of the patients without ETO (*p* = 0.365). The mean time to diagnosis of reinfection following reimplantation was 7.5 months (range, 0.3–15.1 months) in the ETO group compared with 12.5 months (range, 0.7–43 months) in the non-ETO group. Kaplan-Meier survival estimates for reinfection in the ETO group were 87.1% (95% confidence interval [CI]: 81.1–93.1%) at five years with 19 hips at risk and compared with 90.4% (95% CI: 86.7–91.1%) with 39 hips at risk in the non-ETO group (*p* = 0.602).

### 3.3. Aseptic Revision and Other Complications

Four patients (13%) undergoing ETO required aseptic revision after a mean of 5.5 months (range, 0.7–15.0 months) compared to nine patients (14%) without ETO after a mean of 10.9 months (range, 0.7–36.0 months) (*p* = 0.833). Three patients (3.1%) underwent revision for postoperative periprosthetic femoral fractures, all of which occurred in the non-ETO group (*p* = 0.213). One femoral component had to be revised for aseptic loosening in each group. Dislocation occurred in four patients (13%) in the ETO group and eight patients (13%) in the non-ETO group (*p* = 0.978). One patient in each group suffered from traumatic femoral fracture in the prosthesis-free interval and underwent reoperation with stabilization using cerclages and an intramedullary rod. Detailed information on complications is summarized and compared in Table 4. Kaplan-Meier survival estimates for all-cause revision in the ETO group were 74.2% (95% CI: 66.3–82.1%) at five years with 15 hips at risk and compared with 79.4% (95% CI: 74.3–84.5%) with 34 hips at risk in the non-ETO group (*p* = 0.572). 

### 3.4. Radiographic and Clinical Evaluation

The average length of the ETO was 162 mm (range, 98–238 mm). ETO union was observed in 31 (97%) of the 32 hips. In the one patient with non-union, the ETO fragment had been fractured during reduction at the second-stage reimplantation surgery and failed to unite. There were four intraoperative fractures of the ETO fragment (13%), of which all occured at the greater trochanter. Overall, greater trochanter fractures were seen in four hips (13%) of the ETO group and in five hips (8%) of the non-ETO group, respectively (*p* = 0.458). Trochanteric migration of >5 mm was found in 1 (3%) of the 32 hips. Four patients (9%) had a >5 mm femoral stem subsidence in the first three months with subsequent stabilization. No significant differences were detected between the two groups regarding fractures of the greater trochanter and stem subsidence (*p* = 0.458 and *p* = 0.637, respectively). As for the use of prophylactic cerclage at the stage of reimplantation, we found no significant difference with respect to femoral stem subsidence (>5 mm); 2 of 9 hips (22%) vs. 2 of 23 hips (10%) (*p* = *0*.298). Leg-length and offset was restored in the ETO group with a mean postoperative difference of −4.2 mm and −2.6 mm compared to −4.0 mm and −2.6 mm prior the two-stage revision procedure, respectively (*p* > 0.5).

The postoperative improvement in the mHHS score was significant in both groups (*p* < 0.01). mHHS improved from 38 points preceding first-stage surgery to 66 points. The postoperative mHHS did not differ between the ETO and non-ETO group (*p* = 0.700).

## 4. Discussion

ETO has been shown useful for removing well-fixed femoral stems or cement during two-stage revision THA for PJI. Due to the heterogenous operative strategies in the literature, there is still scarce evidence regarding the use of ETO in treating chronic PJI of the hip. It has not yet been finally clarified whether the use of ETO in two-stage revision improves infection eradication compared with two-stage revision without ETO. The optimal method of stem fixation has not been conclusively determined. Finally, the clinical benefits of spacer use in the setting of an ETO, particularly with concomitant bone loss or abductor deficiency, remains unclear. Therefore, the aim of this study was to report on the clinical and radiographic outcome of a consecutive series of ETO without spacer placement during two-stage revision THA for infection.

Our study demonstrated an overall rate of reinfection in patients undergoing ETO of 13% at a mean follow-up of 5.5 years. This is comparable to the existing literature on ETO in two-stage revision THA, ranging from 3% to 23% [18,19,20,21,22]. Only one study has compared two-stage revision with and without ETO for the management of hip PJI and demonstrated improved infection-free rates in ETO cases [22]. In our matched-control study, we did not find such difference in infection eradication following THA reimplantation. However, patients undergoing ETO had a significantly lower rate of repeated debridement following first-stage implant removal, which is in accordance with the series of Shi et al. [22]. A rationale behind improved infection eradication in the setting of an ETO is that it allows a more thorough debridement and cement removal due to a much better visualization of the femoral cavity. However, following THA reimplantation, reinfection rates were similar in the ETO and non-ETO groups in our series. There are several possible explanations. In the underlying study, patients who underwent ETO had a significantly worse femoral bone stock prior first-stage surgery than patients in the non-ETO group. Insufficient debridement of possible devitalized bone with biofilm residues [43] might have contributed to reinfection, which was not apparent in the interim period but later following reimplantation. In the ETO group, patients had a worsening local extremity status prior treatment. It is still unclear whether changes in the vascularization of the soft tissue affect immune responses [44]. Patients more often presented with a sinus tract in the ETO group, which might have adversely affected the outcome [45]. Finally, all reinfections in the ETO group showed new pathogens, which might represent new infections introduced at reimplantation or be due to hematogenous spreading [46].

In the underlying study, the ETO union rate was 97%, which is in line with previous studies, which ranged from 96% to 100% [18,19,20,21,22]. These findings demonstrate that ETO with immediate cerclage fixation in the presence of PJI does not compromise osteotomy healing. The potential concern of compromising infection eradication due to metallic hardware to fix the ETO fragment seems to play a negligible role, especially considering that cerclages are exchanged or removed at the time of reimplantation. The placement of a prophylactic cable distal to the ETO has been advocated for in an attempt to decrease the risk of intraoperative fracture during stem insertion [47]. In the underlying study, no intraoperative diaphyseal fracture was noticed regardless of the use of a prophylactic cable and all three postoperative fractures occurred in the non-ETO group. Another reason to use a prophylactic diaphyseal cerclage might be to achieve better press-fit during broaching to mitigate stem subsidence. Interestingly, however, in our series there was no difference in stem subsidence rates between ETOs with and without prophylactic cerclages.

We observed an intraoperative fracture rate of 13%, which is in line with the literature [18,19,20,21,22]. All intraoperative fractures occurred at the portion of the greater trochanter mostly due to an osteolytic ETO fragment itself. Intraoperative fractures may be avoided by keeping the ETO fragment wide, using a pencil-tip burr to facilitate a gentle elevation, and being cautious while handling and fixing the ETO fragment. Postoperative femoral fractures were seen in three patients (3%), all of which occurred in non-ETO patients. This trend towards a higher risk of postoperative fractures may be explained by the fact that endofemoral stem removal may not be performed consistently, resulting in unnoticeable weakening of the femoral cortex.

The dislocation rate was 12.5% in our series and there was no difference between the ETO and non-ETO group. High rates of dislocation following ETO in two-stage revision THA have been shown, ranging from 4% to 31% [18,19,20,21,22]. These high rates of instability do not appear to correlate with the use of ETO, as instability represents the most common complication with similar rates in two-stage exchange THA without ETO [7,8,12,48]. Our recommendation is to maximally preserve the remaining abductor mechanism while carefully positioning the components, readjusting the ETO fragment carefully, and using large diameter heads and a dual-mobility cups. 

The main rationale for performing ETOs without spacers is to mitigate the high risk of spacer-associated complications, with reported rates of up to 25% [18,19,20,21,22,29]. In our series, only one patient (3%) with ETO required revision for a fracture in the interim period. Therefore, a nonspacer ETO might represent a safer alternative in two-stage revision THA, particularly in cases with additional bone loss and abductor deficiency. However, a concern of not using hip spacers is limb shortening and muscle contracture making reimplantation technically more challenging, especially leg-length restoration. In the underlying series, leg-length was successfully restored to the leg-length that was present before the treatment, and clinical outcome scores measured with the mHHS improved significantly to values that are similar to studies reporting on ETO in two-stage exchange THA with spacer placement [18,19,20,21,22].

In our series, only cementless porous-coated, diaphyseal-engaging stems were utilized in ETO cases. In more than one-third of the cases, rectangular tapered revision stems were used for reimplantation, provided that the ETO length and healing allowed a stable fixation. Overall, stem stability was excellent, with only one patient requiring revision for aseptic loosening. In this case, an undersized rectangular tapered stem in a hip with a healed osteotomy led to early loosening and therefore failure was independent of the ETO. Petrie et al. [19] have advocated for cemented standard-length stems in patients with successful ETO union, whereas the authors in this study chose cementless long stems in cases with proximal femoral bone defects. We believe that cementless femoral components should be prioritized whenever possible. In case of reinfection, adequate removal of cemented femoral components can be very challenging and may further compromise the femur distally. However, there is still a dearth in the literature and the ideal type of stem fixation in terms of both implant survivorship and difficulty when facing septic failure remains controversial.

There are several limitations to the current study. First, this was a retrospective analysis, which has inherent drawbacks. Second, given our relatively small number of ETO cases, we were unable to examine the influence of other confounding variables. We compensated for this limitation by performing a matched cohort analysis with twice as many non-ETO patients. Third, despite using a standardized two-stage protocol, several variables had minor variations, including the types of prosthesis removed, degree of debridement, length of interim period and implant selection for reimplantation.

## 5. Conclusions

This study shows that ETO with intermediate resection arthroplasty is a safe and effective method to manage patients with well-fixed femoral stems and for thorough cement removal in two-stage revision THA for PJI. Based on the currently available data, the use of ETO results in comparable, if not superior, infection control compared to two-stage revision without ETO. A nonspacer regimen and the absence of local antibiotics does not seem to compromise infection eradication rates. ETO nonunion and clinically important trochanteric migration are rare. Cementless long-stemmed femoral components showed good survivorship at mid-term, with comparable functional outcomes and leg-length restoration. Further larger-scale studies are required to investigate the impact of hip spacers in the setting of ETO on the clinical outcomes of two-stage revision THA for PJI.

## Figures and Tables

**Table 1:** Comparison of operative characteristics between ETO and non-ETO group.

Variable	ETO (*n* = 32)	Non-ETO (*n* = 64)	*p* Value
Cementation in earlier prosthesis	4 (13%)	18 (28%)	0.086
Time from index THA (years)	7.8 ± 6.7	7.9 ± 8.3	0.932
Paprosky bone loss, femoral			0.001
1	5 (16%)	29 (45%)	
2	12 (38%)	26 (41%)	
3A	9 (28%)	4 (6%)	
3B	6 (19%)	3 (5%)	
4	0 (0%)	2 (3%)	
Paprosky bone loss, acetabular			0.639
1	2 (6%)	8 (13%)	
2A	6 (19%)	14(22%)	
2B	3 (9%)	7 (11%)	
2C	12 (38%)	25 (39%)	
3A	3 (9%)	6 (9%)	
3B	5 (16%)	3 (5%)	
Pelvic discontinuity	1 (3%)	1 (2%)	
Duration of first-stage surgery (minutes)	192.8 ± 58.6	143.3 ± 51.6	0.000
ETO length (mm)	162 (98–238)	-	-
Reimplanted components at second-stage			
Femoral			
Extensively porous-coated	13 (41%)	51 (80%)	0.000
Modular, fluted tapered	19 (59%)	9 (14%)	
Cemented	0 (0%)	4 (6%)	
Acetabular			0.953
Modular, porous-coated	3 (9%)	8 (13%)	
Highly porous Trabecular metal	20 (63%)	40 (63%)	
Antiprotrusio cage	5 (16%)	8 (13%)	
Cemented	4 (13%)	8 (13%)	
Dual-mobility articulation	5 (16%)	10 (16%)	1.000
Duration of second-stage surgery (minutes)	169.3 ± 47.9	145.3 ± 57.7	0.046

Means and standard deviations are reported, and *p* values were calculated either from chi-square test or Mann–Whitney U test. ETO, extended trochanteric osteotomy; THA, total hip arthroplasty.

**Table 2:** Comparison of microorganism frequency between ETO and non-ETO group.

Isolated Microorganism *	ETO (*n* = 32)	Non-ETO (*n* = 64)
Gram-positive bacteria		
Coagulase-negative *Staphylococcus* (sensitive)	12	34
Coagulase-negative *Staphylococcus* (resistant)	1	0
Methicillin-sensitive *Staphylococcus aureus*	4	11
Methicillin-resistant *Staphylococcus aureus*	0	3
*Cutibacterium* spp.	3	5
*Staphylococcus lugdunensis*	1	1
Viridans group *Streptococcus*	1	2
Entercoccus faecalis	2	3
Enterococcus faecium	0	3
Peptostreptococcus micros	1	2
Finegoldia magna	3	1
*Corynebacterium* spp.	0	2
*Actinomyces* spp.	0	2
*Peptoniphilus* spp.	1	2
Cellulomonas	0	1
Gram-negative bacteria		
*Escherichia coli*	1	4
*Roseomonas mucosa*	0	1
Polymicrobial	5	25
Negative culture	3	7

* Includes preoperative and intraoperative cultures during first-stage surgery.

**Table 3:** Comparison of demographic data between ETO and non-ETO groups.

Variable	ETO (*n* = 32)	Non-ETO (*n* = 64)	*p* Value
Age at first-stage (years)	71.3 ± 10.5	70.9 ± 7.3	-
Sex (M:F)	11:21	22:42	-
BMI (kg/m2)	27.1 ± 5.2	29.3 ± 5.4	0.076
Systemic host grade			0.291
A	9 (28%)	10 (16%)	
B	17 (53%)	36 (56%)	
C	6 (19%)	18 (28%)	
Local extremity grade			0.147
II	23 (72%)	54 (84%)	
III	9 (28%)	10 (16%)	
Sinus tract present	8 (25%)	8 (13%)	0.121
Microbiology at first-stage			
Difficult-to-treat *	6 (19%)	9 (14%)	0.551
Negative cultures	3 (9%)	7 (11%)	0.813
Positive cultures at second-stage	3 (9%)	6 (9%)	1.000
Weeks between stages	8.8 ± 5.4	9.1 ± 4.3	0.712
Follow-up (months)	66.1 ± 20.0	65.5 ± 17.4	0.882

Means and standard deviations are reported, and p values were calculated either from chi-square test or Mann–Whitney U test. ETO, extended trochanteric osteotomy; BMI, body mass index. * Pathogens, for which no biofilm-active antibiotics exist (rifampin-resistant staphylococci, enterococci, ciprofloxacin-resistant Gram-negative bacteria and fungi).

**Table 4:** Comparison of radiographic results, complications and functional outcome between ETO and non-ETO group.

	ETO (*n* = 32)	Non-ETO (*n* = 64)	*p* Value
Radiographic results			
ETO fragment fracture	4 (13%)	5 (8%) *	0.458
ETO migration (>5 mm)	1 (3%)	-	
Union of ETO	31 (97%)	-	
Femoral stem subsidence (>5 mm)	4 (13%)	6 (9%)	0.637
Reinfection			
Interim re-debridement for infection persistence	0 (0%)	9 (14%)	0.026
Reinfection after reimplantation	4 (13%)	6 (9%)	0.365
Other complications			
Traumatic femoral fracture in the interim period	1 (3%)	1 (2%)	0.613
Early superficial wound complication after first-stage	2 (6%)	4 (6%)	1.000
Early superficial wound complication after reimplantation	1 (3%)	7 (11%)	0.192
Hip instability after reimplantation	4 (13%)	8 (13%)	0.978
Periprosthetic femoral fracture after reimplantation	0 (0%)	3 (5%)	0.213
Cup loosening	0 (0%)	1 (2%)	0.477
Stem loosening	1 (3%)	1 (2%)	0.613
Functional outcome			
Preoperative mHHS before first-stage	37.7 ± 17.1	37.3 ± 12.2	0.904
Postoperative mHHS at final follow-up	65.9 ± 15.7	67.4 ± 15.2	0.700

Means and standard deviations are reported. ETO, extended trochanteric osteotomy; mHHS, modified Harris hip score. * Greater trochanter fractures.

## Data Availability

The data presented in this study are available upon reasonable request from the corresponding author.
